# Robust analysis of a novel PANoptosis-related prognostic gene signature model for hepatocellular carcinoma immune infiltration and therapeutic response

**DOI:** 10.1038/s41598-023-41670-9

**Published:** 2023-09-04

**Authors:** Yongguang Wei, Chenlu Lan, Chengkun Yang, Xiwen Liao, Xin Zhou, Xinlei Huang, Haixiang Xie, Guangzhi Zhu, Tao Peng

**Affiliations:** 1 Department of Hepatobiliary Surgery, The First Affiliated Hospital of Guangxi Medical University, Nanning, 530021 China; 2 Guangxi Key Laboratory of Enhanced Recovery After Surgery for Gastrointestinal Cancer, 530021 Nanning, People’s Republic of China; 3 Key Laboratory of High-Incidence-Tumor Prevention and Treatment (Guangxi Medical University), Ministry of Education, Nanning, 530021 China

**Keywords:** Cancer, Computational biology and bioinformatics, Drug discovery, Immunology

## Abstract

PANoptosis, an interplay between pyroptosis, apoptosis, and necroptosis, is deeply involved in cancer development and immunity. However, the influence of PANoptosis in hepatocellular carcinoma (HCC) remains to be further investigated. The differentially expressed PANoptosis-related genes (PANRGs) was screened in The Cancer Genome Atlas (TCGA) database. Accordingly, mutation, bioinformatics, and consensus clustering analyses were performed. Then, a prognostic risk model was developed by least absolute shrinkage and selection operator (LASSO) Cox regression. Furthermore, the prognostic value, immunity correlation and therapeutic response prediction ability of risk model were explored. A total of 18 PANRGs were differently expressed in the TCGA-HCC cohort and were mainly involved in cancer- and cell death-related signal pathways. Using unsupervised clustering method, we identified two PANRGs-mediated clustering patterns. The remarkable differences between the two clusters on overall survival (OS) and clinical features were demonstrated respectively. Based on the five-gene prognostic risk model, the calculated PANRG-scores were used to categorize the subgroups as high- and low-risk. Notably, the high-risk subgroup had a dismal prognosis and exhibited much lower immune infiltration levels of mast cells, nature killer cells and pDCs, but higher levels of aDCs, iDCs and Treg cells than those in the low-risk subgroup. Furthermore, we constructed a reliable nomogram combining clinical traits and PANRG-score to predict the OS of HCC patients. The significantly negative correlation between PANoptosis and tumor mutation burden (TMB), ferroptosis were revealed. In drug sensitivity analysis, the high-risk subgroup had a considerably lower TIDE score, suggesting a preferable response to immunotherapy, and may be more sensitive to Tipifarnib, Imatinib, Doxorubicin, and Gemcitabine. The upregulated mRNA expressions of *FADD* were validated in 16 paired HCC tissues of Guangxi cohort. Based on PANoptosis-related genes, an integrated risk signature was constructed to provide a roadmap for patient stratification and predict HCC patient's prognosis. The patients with the higher PANRG-score may carry a dismal survival and relatively low immune infiltration, but a potential better immunotherapy response. Therefore, future HCC therapy perspectives should emphasize the setting of PANoptosis to achieve a personalized, practicable and effective therapeutic regimen.

## Introduction

PANoptosis, a newly discovered cell death pathway with morphological and biochemical properties, highlights the interface between the three Programmed cell death (PCD) pathways, namely pyroptosis, apoptosis, and necroptosis. Substantial evidence has shown PANoptosis, attaching importance to infectious or metabolic diseases, autoimmunity and cancer, is an interlace of immune response^1–6^. In recent years, emerging molecular targeted agents and immunotherapy display a great application prospect in systematic therapy of liver cancer. PCD enables elimination of modified cancer cells to impede cancer progression through the patients’ own immune system^7^. Immunotherapy, particularly immune checkpoint inhibitor, efficiently triggered the immune system to attack tumor cells by hindering immune escape. Nevertheless, drug resistance and progressive disease during immunotherapy really make us frustrated and they may be associated with resistance to drug-induced cell death^8^. Thus, more researches on seeking in-depth understanding of primary PCD types, including PANoptosis, should be performed to overcome cell death-related drug resistance, improve medication sensitivity and develop customized therapy regimens for HCC patients.

Hepatocellular carcinoma (HCC) accounts for 85%-90% of primary liver cancer cases and is a prevalent malignant tumor of the digestive system worldwide^9^. In contemporary clinical practice, chronic viral hepatitis and alcoholic or nonalcoholic fatty liver disease are the dominating etiologies of HCC^9, 10^. Since HCC frequently develops asymptomatically, most patients were already in a late stage when they were initially diagnosed, meaning they had missed the chance of curative resection. Despite the ongoing therapeutic strategy improvement, the long-term survival of HCC patients is still low^11^, which exerts a heavy burden on society^9^. According to a retrospective study, 52%, 18%, and 9% of patients survived at 5-, 10-, and 15-years respectively after having hepatic resection ^12^. Cell death is a crucial field in the genesis and development of liver carcinogenesis. Under specific circumstances, interactive model of PANoptosis enables different cell death pathways to change from one mode to another. The primary controlling regulators of PANoptosis have been identified as PANoptosomes, a cytoplasmic polymeric protein complex^5, 13, 14^. Acting as focal points, PANoptosomes induces mutiple types of cell death, mainly including pyroptosis, apoptosis, and necroptosis^5, 15^. It is demonstrated that PANoptosomes can cause viral-induced fulminant hepatitis in mice by activating the coronavirus murine hepatitis virus^16, 17^. During the viral infection, inflammatory PANoptosis can be triggered to offer host protection when *AIM2* controls the innate immune sensors pyrin and *ZBP1*^18^. Conversely, *ADAR1* may inhibit PANoptosis mediated by *ZBP1*, which drives carcinogenesis^19^. Therefore, in contrast to the typically detrimental effects of an inflammatory state, PANoptosis may be beneficial to cancer^20, 21^. For example, *TNF*- and *IFN*-induced PANoptosis creates a lethal mechanism to thwart the progression of cancer^22^. Moreover, PANoptosis induced by combined therapies of metformin and doxorubicin can inhibit the melanoma cells from progressing *in vitro* and *in* *vivo*^23^. Besides, the researchers have revealed that the PANoptosis-related grading system also makes sense to gastrointestinal cancer^24, 25^.

Currently, for HCC with dismal prognosis, the study on cellular and molecular mechanism of its carcinogenesis and progression is worth carrying out in depth. More studies are still needed to clearly comprehend the specific involvement that PANoptosis plays in the evolution of HCC. Therefore, we concentrate on the prognostic and therapeutic significance of PANoptosis gene signatures in the present study. Robust analysis was carried out to identify differently expressed and prognostic PANRGs which contributed to construct a PANRG-score for HCC patients. Ultimately, the PANRG-score makes sense in terms of prognostic risk stratification, tumor immunoinfiltration evaluation, and therapeutic response prediction.

## Results

### Identification of differentially expressed PANRGs

**Figure 1:**
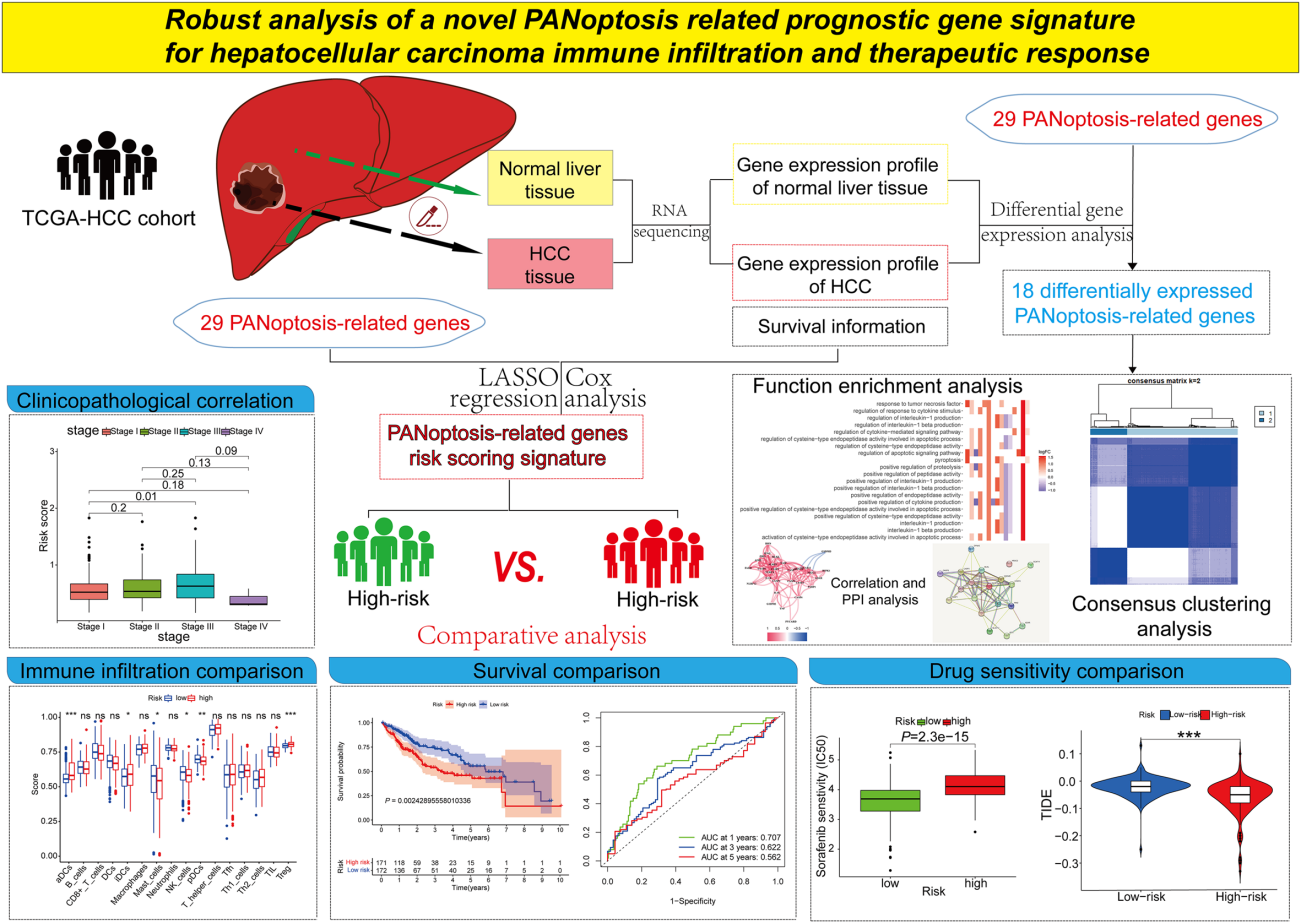
The graphical abstract showed the flow chart of methods and the main results in our study.

**Figure 2:**
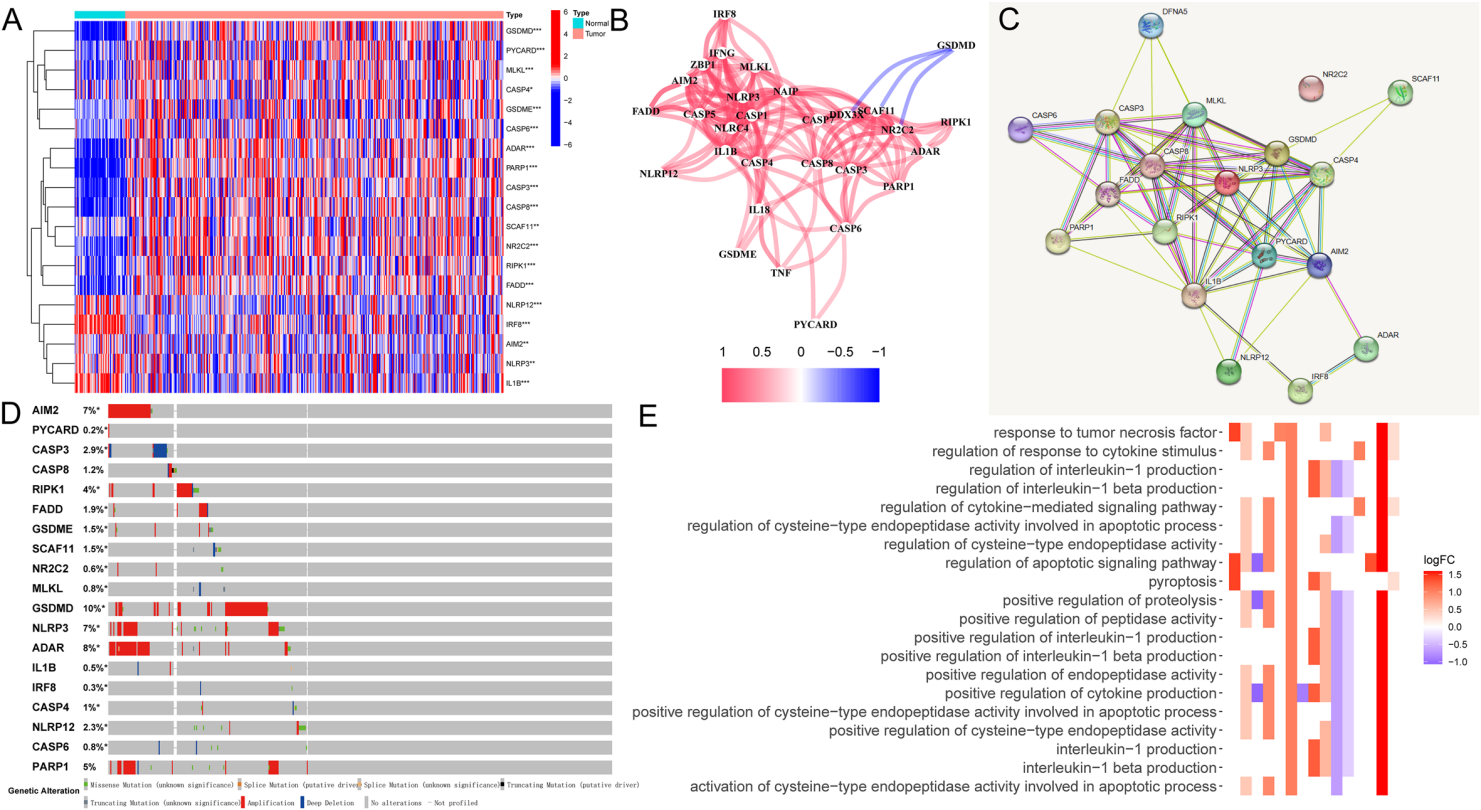
**(A**) A heatmap highlighted that a total of 18 PANRGs were differently expressed between normal and HCC samples in the TCGA-HCC cohort. Red indicates genes with a high expression level, while blue indicates the low expression level. *P < 0.05, ** *P* < 0.01, *** *P* < 0.001. (**B**) The correlation network showed the positive correlations among PANRGs except *GSDMD* (red line, positive correlation; blue line, negative correlation. The intensity of the colors represents the degree of the relevance). (**C**) A PPI network displayed the interconnections of the differentially expressed PANRGs, in which *FADD* was one of the hub genes. (**D**) The mutation frequency and classification of 18 differentially expressed PANRGs were explored in cBioPortal, in which *GSDMD* being the gene with the highest mutation rate. (**E**) The enriched items in gene ontology analysis showed the differentially expressed PANRGs were significantly correlated with cell death- and cytokine regulation-related pathways. Red indicates positive correlation, while blue indicates negative correlation. The intensity of the colors represents the strength of relevance. PANRGs, PANoptosis-related genes; TCGA, the Cancer Genome Atlas database; HCC, hepatocellular carcinoma; PPI, protein–protein interactions.

The graphical abstract containing the flow chart of methods and the main results in our study was showed in Fig. 1A total of 29 PANoptosis-related genes (PANRGs) presented in Supplementary Table S1 were identified from the previous literatures. Using differential gene expression analysis in TCGA-HCC cohort, a heatmap illustrated that a total of 18 PANRGs (*AIM2, PYCARD, CASP3, CASP8, RIPK1, FADD, GSDME, SCAF11, NR2C2, GSDMD, NLRP3, ADAR, IL1B, IRF8, CASP4, NLRP12, CASP6, PARP1*) were differentially expressed between HCC and normal liver tissues (Fig. 2A). The distributions of PANRGs copy number variations (CNV) alterations on chromosomes were shown on a Circos graph (Supplementary Fig. 1A). The correlation network that was comprised of all the PANRGs showed the positive correlations among every PANRGs except *GSDMD* (Fig. 2B). The protein–protein interactions (PPI) network of differently expressed PANRGs was fashioned in STRING and *FADD, RIPK1, PYCARD, IL1B, CASP4, CASP3, NLRP3, CASP8, MLKL* and *AIM2* were determined as the hub genes of network (Fig. 2C).

### Landscape of genetic variation of differentially expressed PANRGs.

To explore the genetic variation landscape of HCC, we summarized the overall incidence and frequency of CNV and somatic mutations based on the TCGA-HCC dataset. The results suggested that the main variant classification was missense mutation and the most frequent variant type was single nucleotide polymorphisms (SNP). Base transition C to A ranked as the chief single nucleotide variants (SNV) occurrence class (Supplementary Fig. 1B). In addition, in terms of the genetic alterations and the mutation frequencies of the differentially expressed PANRGs, the results from cBioPortal website showed that six genes had a mutation rate ≥ 3%, in which *GSDMD* was the gene with the highest mutation rate of 10% (Fig. 2D).

### Functional enrichment analysis of differentially expressed PANRGs

The gene ontology (GO) enrichment analysis revealed that the 18 differentially expressed PANRGs were significantly correlated with “pyroptosis, regulation of interleukin-1 production, response to tumor necrosis factor, positive regulation of cytokine production, apoptotic process, regulation of apoptotic signaling pathway, and regulation of cytokine − mediated signaling pathway” (Fig. 1E). The Kyoto encyclopedia of genes and genomes (KEGG) enrichment analysis demonstrated that they primarily participated in the HCC-related pathways, such as “Toll-like receptor signaling pathway, TNF signaling pathway, NOD-like receptor signaling pathway, Apoptosis, Hepatitis C, C-type lectin receptor signaling pathway, IL-17 signaling pathway, Cytosolic DNA-sensing pathway, and Alcoholic liver disease” signaling pathways (Supplementary Fig. 1C). These results suggested that PANoptosis may develop an underlying regulatory mechanism to influence HCC carcinogenesis, cell death and liver diseases.

### Identification of the PANRGs-mediated HCC classification patterns

**Figure 3:**
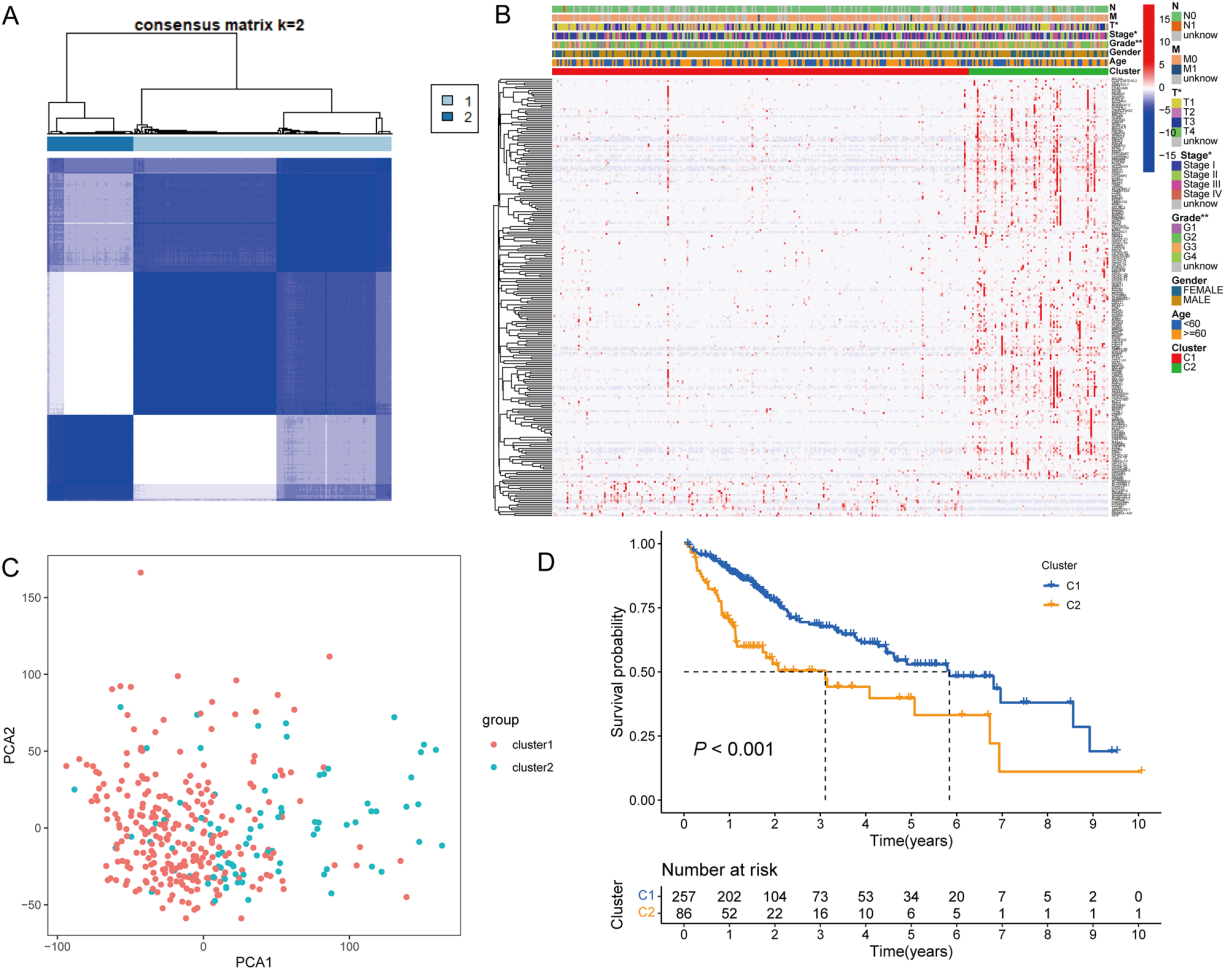
(**A**) Based on the PANRGs expression level in TCGA-HCC cohort, two PANoptosis-related clusters were identified by consensus clustering analysis. (**B**) The heatmap presented the gene expression profile of differentially expressed genes in the TCGA-HCC dataset and illustrated that the clinicopathological factors, such as T staging, pathological grade and clinical TNM stage were significantly different between the two clusters. *P < 0.05, ** *P* < 0.01, *** *P* < 0.001. (**C**) The independence of the two clusters in the TCGA-HCC dataset was established in principal component analysis. (**D**) Kaplan–Meier OS curves for the two clusters in the TCGA-HCC dataset demonstrated that Cluster 1 had a significant survival advantage over Cluster 2. PANRGs, PANoptosis-related genes; TCGA, the Cancer Genome Atlas database; HCC, hepatocellular carcinoma; TNM; tumor, node and metastasis; OS, overall survival.

Based on the unsupervised clustering approach and the expression profiles of PANRGs in the TCGA-HCC dataset, two distinct regulation patterns were identified (Fig. 3A). The heatmap presented not only the gene expression profile of differentially expressed genes but also depicted the significantly different clinicopathological factors, such as T staging, pathological grade and clinical TNM stage between cluster 1 and 2 (Fig. 3B). In the principal component analysis (PCA) analysis, two PANRGs-mediated patterns could be categorized (Fig. 3C). The PANRGs-mediated cluster 1 (C1) incorporated 257 cases, while cluster 2 (C2) incorporated 86 cases and C1 has a significant survival advantage over C2 (Fig. 3D). In conclusion, the PANRGs-mediated HCC cluster pattern carried a favorable stratification ability of tumor stage and prognostic risk.

### Development of a PANRGs-related prognostic risk signature

**Figure 4:**
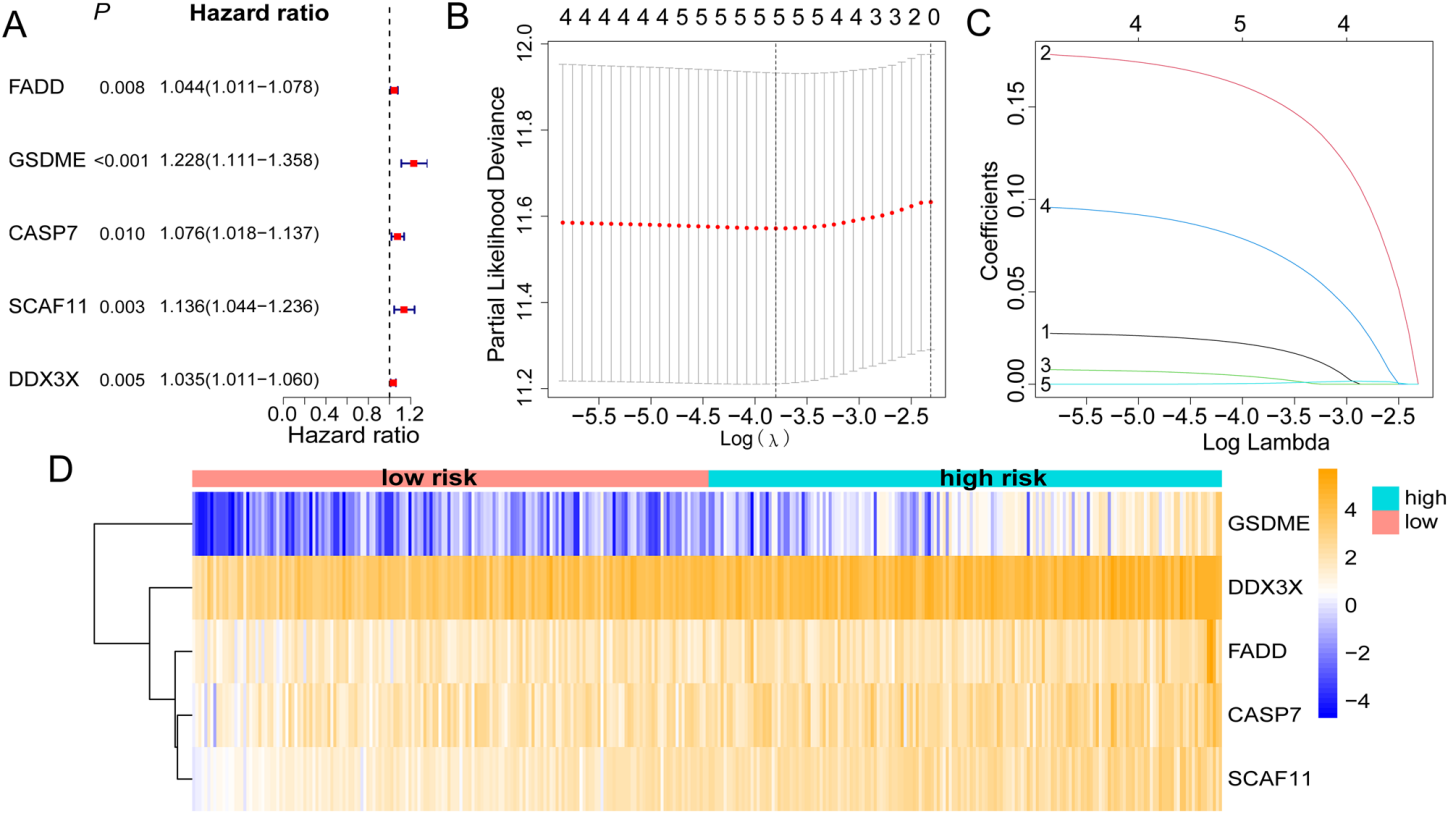
(**A**) Forest plot illustrated the result of univariate Cox regression analysis in TCGA-HCC dataset, in which five candidate genes were screened preliminarily (all *P* < 0.05). (**B**,**C**) LASSO regression analysis was utilized to further diminish dimensionality and formulate the prognostic risk model based on the TCGA-HCC dataset. (**D**) Heatmap showed the expression profile of five genes in PANRGs-related prognostic risk signature between the high- and low-risk subgroups. TCGA, the Cancer Genome Atlas database; HCC, hepatocellular carcinoma; LASSO, least absolute shrinkage and selection operator; PANRGs, PANoptosis-related genes.

**Figure 5:**
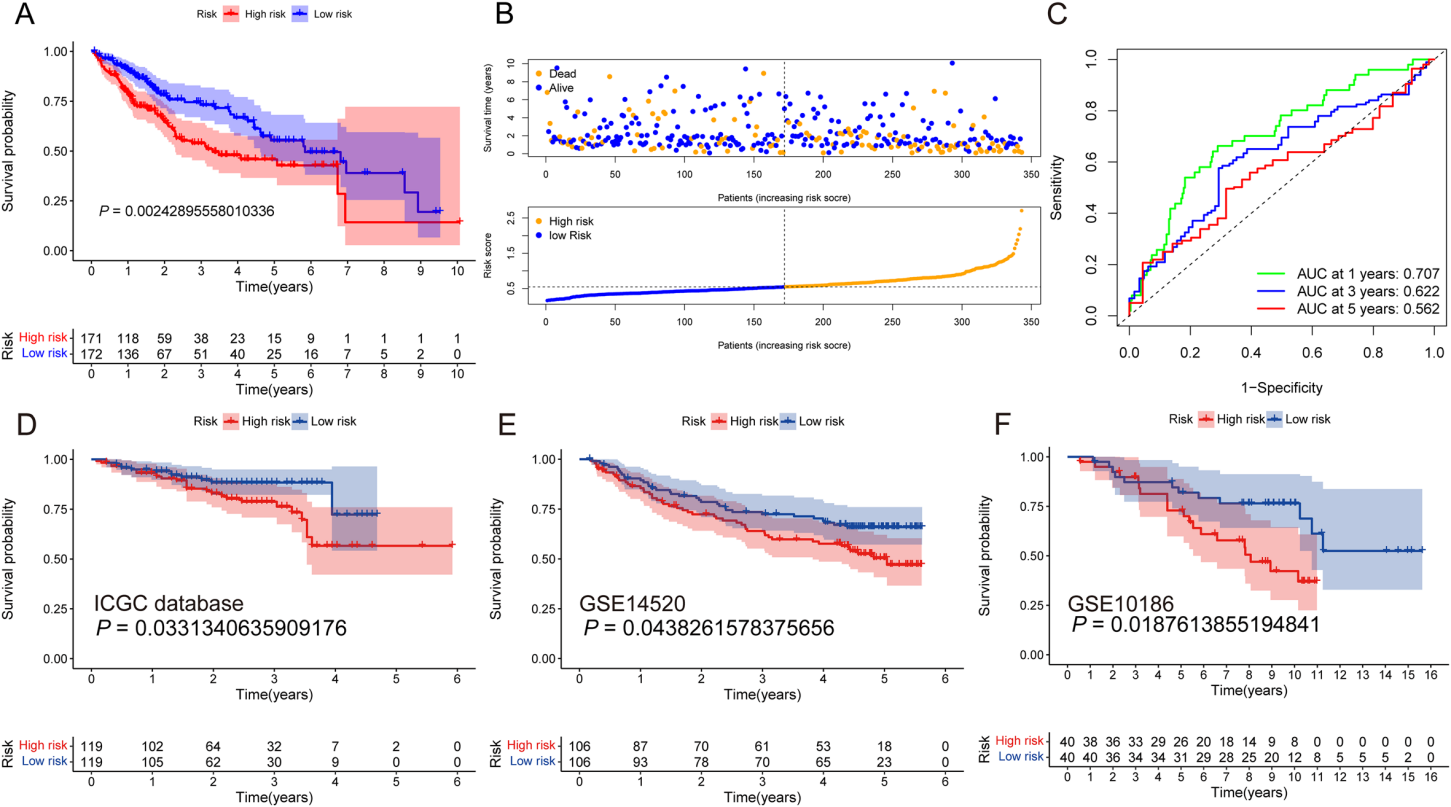
(**A**) The Kaplan–Meier curve between the high- and low-risk subgroups in TCGA-HCC corhort showed that the high-risk subgroup took on a significantly shorter OS. (**B**) Scatter plots of TCGA-HCC patient distributions based on the different PANRG-scores showed that high-risk subgroup had a greater mortality risk. (**C**) ROC curves demonstrated the decent prediction efficiency of the PANRG-score for 1-, 3- and 5-year OS. (**D**–**F**) Kaplan–Meier curves for the OS in the ICGC-HCC (**D**), GSE14520 (**E**), and GSE10186 (**F**) cohorts further validated the prognostic value of PANoptosis-related risk scoring system (all *P* < 0.05). TCGA, the Cancer Genome Atlas database; HCC, hepatocellular carcinoma; OS, overall survival; PANRG, PANoptosis-related gene; ROC, receiver operating characteristic curve; ICGC, International Cancer Genome Consortium.

On condition that both value of *P* and KM < 0.05, a total of 5 candidate gene signatures were screened from PANRGs utilizing univariate analysis (Fig. 4A). After being further refined by the least absolute shrinkage and selection operator (LASSO) Cox regression analysis (Fig. 4B,C), a PANRGs-related prognostic risk signature was ultimately established. The formula was developed: PANRG-score = (0.020 × *FADD*) + (0.157 × *GSDME*) + (0.036 × *CASP7*) + (0.074 × *SCAF11*) + (0.0004 × *DDX3X*). Based on the median of calculated risk score, a total of 343 TCGA-HCC patients were categorized equally into high- and low-risk groupings and the heatmap showed the expression profile of five key genes between the different subgroups (Fig. 4D), in which *FADD*, *GSDME* and *SCAF11* were the differentially expressed PANRGs (Figs. 2A and 4D). The survival analysis demonstrated the high-risk subgroup took on a significantly shorter overall survival (OS) period and a greater mortality risk compared to the low-risk group (Fig. 5A,B). Receiver operating characteristic (ROC) analysis manifested that the area under curve (AUC) values were 0.707 for 1-year, 0.622 for 3-year, and 0.562 for 5-year OS respectively, which proved a promising survival prediction ability of our PANRG-score signature (Fig. 5C). Actually, the prognostic ability of PANRG-score alone was limited and combination with other clinical parameters, such as tumor stage and differentiated degree, should be considered. Additionally, the reliable clustering ability of signature was demonstrated by principal component analysis (PCA) and t-Distributed Stochastic Neighbor Embedding (t-SNE) analysis in TCGA and ICGC cohorts (Supplementary Fig. 2A–D).

### Validated the prognosis value and biological functions of the PANRGs-related signature model

Furthermore, the Kaplan–Meier survival analyses in the validation datasets highlighted the prognostic ability of PANRG-score model ulteriorly and verified the worse prognosis in the high-risk subgroup (Fig. 5D–F). Using Gene Set Enrichment Analysis (GSEA) software program, we discovered multiple cell death- and cancer-related pathways such as “cell kill, JAK-STAT signal pathway, T cell and nature killer cell mediated cytotoxicity, VEGF signal pathway, and B cell, T cell and Toll like receptor signal pathway” were considerably abundant in the low-risk subgroup (Supplementary Fig. 3).

### Building a predictive nomogram

**Figure 6:**
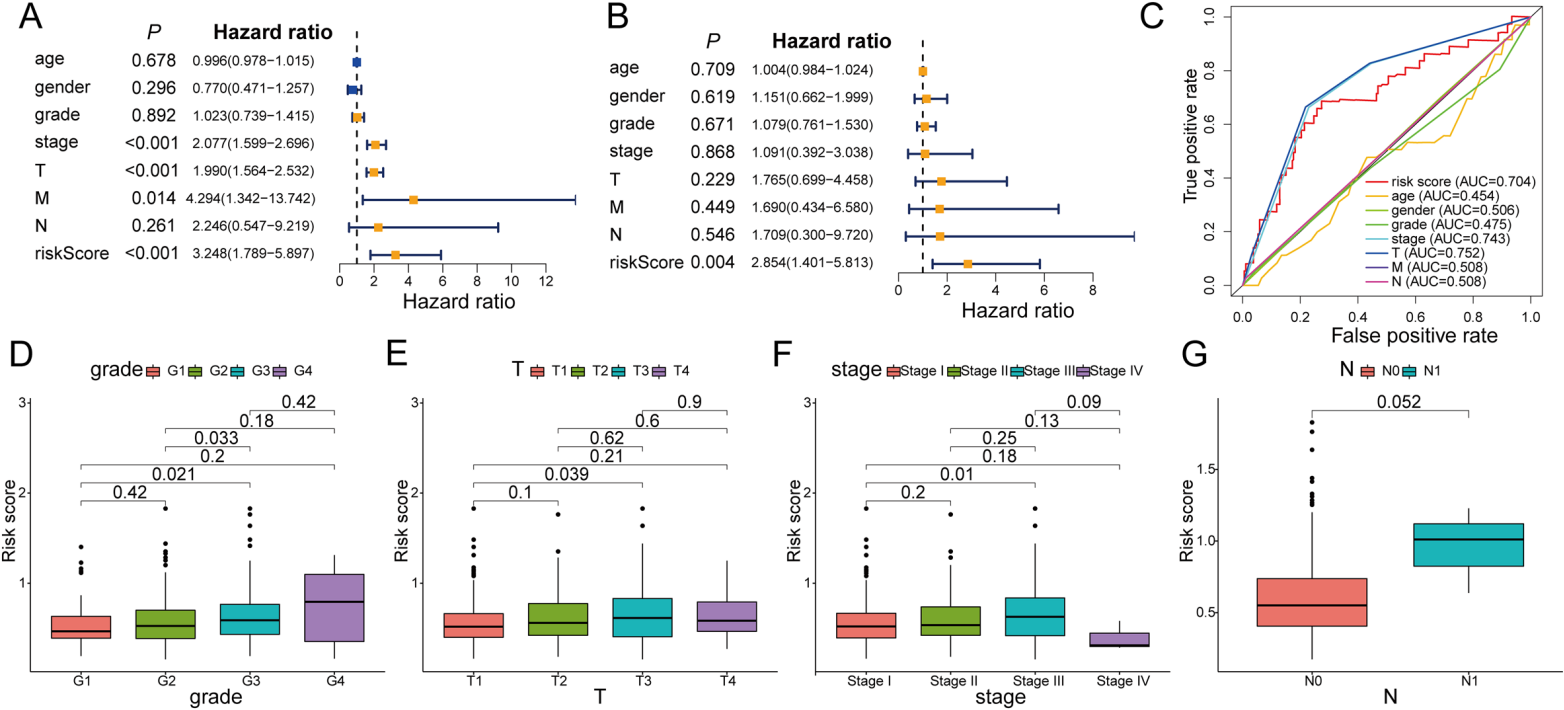
(**A**,**B**) Forest plot of univariate and multivariate Cox regression analyses for the PANRG-score and clinicopathologic parameters demonstrated that PANRG-score was a reliable OS predictor of TCGA-HCC patients. (**C**) The muti-ROC curves illustrated that PANRG-score, compared with other clinicopathological factors, had a preferable prognostic value for TCGA-HCC patients. (**D**–**G**) The PANRGs-related risk signature was stratified by clinicopathological factors including pathological grade, T stage, tumor stage and lymph node metastasis status in the TCGA-HCC cohorts. PANRG, PANoptosis-related gene; OS, overall survival; TCGA, the Cancer Genome Atlas database; HCC, hepatocellular carcinoma.

The results of univariate and multivariate Cox regression analyses were exhibited in a forest map (Fig. 6A,B), in which PANRG-score was a reliable OS predictor of HCC patients (HR = 2.854, *P* < 0.05). The muti-ROC analysis suggested PANRG-score with the AUC value of 0.704 had a preferable prognostic value for HCC patients compared with other clinicopathological factors (Fig. 6C). Furthermore, the PANRG-scores were remarkably different between the various clinical stages (T-stage1 *vs.* T-stage 3; Stage I *vs.* Stage II) and pathological grades (Grade1 vs. Grade3). Hence, the PANRG-score risk model could be predictive of tumor growth and malignant degree (Fig. 6D–G). To comprehend the impact on prognosis of HCC patients, a nomogram featuring PANRG-score and clinicopathological characteristics was drawn for the prediction of 1-, 3-, and 5-year OS and its C-index is 0.725 (Fig. Supplementary 4A). Moreover, the calibration curves illustrated that the comprehensive nomogram model carried an accurate predictive effectiveness of early prognosis (Fig. Supplementary 4B,C). To make manipulation and usage easier, the nomogram was adapted into a web-based calculator, by which we can enter or select the specific value of clinical parameter and then the predicted survival of HCC patient can be showed correspondingly (https://yongguangwei.shinyapps.io/DynNomapp/; Supplemental Fig. 5).

### Immune-related, TMB, and drug-sensitivity analysis of PANRGs

**Figure 7:**
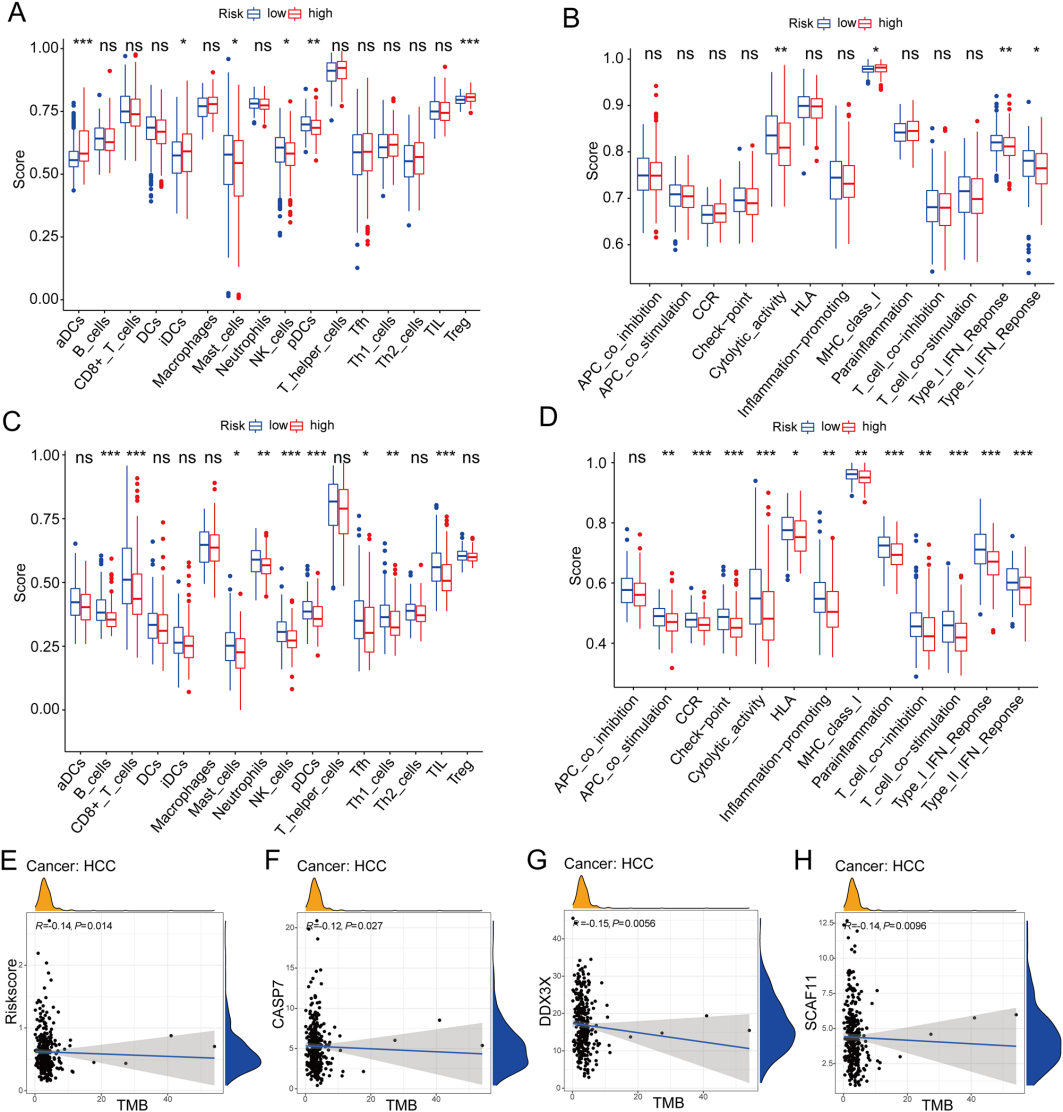
(**A**–**D**) In terms of 13 immune-related functions and the 16 types of immune infiltration cells, Comparison of their enrichment scores the between the high- and low-risk subgroups were conducted in the TCGA (**A**,**B**) and ICGC (**C**,**D**) cohorts respectively. *P < 0.05, ** *P* < 0.01, *** *P* < 0.001. (E–H) The negative correlations were revealed between the TMB and PANRG-score, *DDX3X*, *CASP7*, *GSDME* expression levels in TCGA-HCC dataset. ICGC, International Cancer Genome Consortium; TMB; Tumor Mutation Burden; PANRG, PANoptosis-related gene; TCGA, the Cancer Genome Atlas database; HCC, hepatocellular carcinoma.

**Figure 8:**
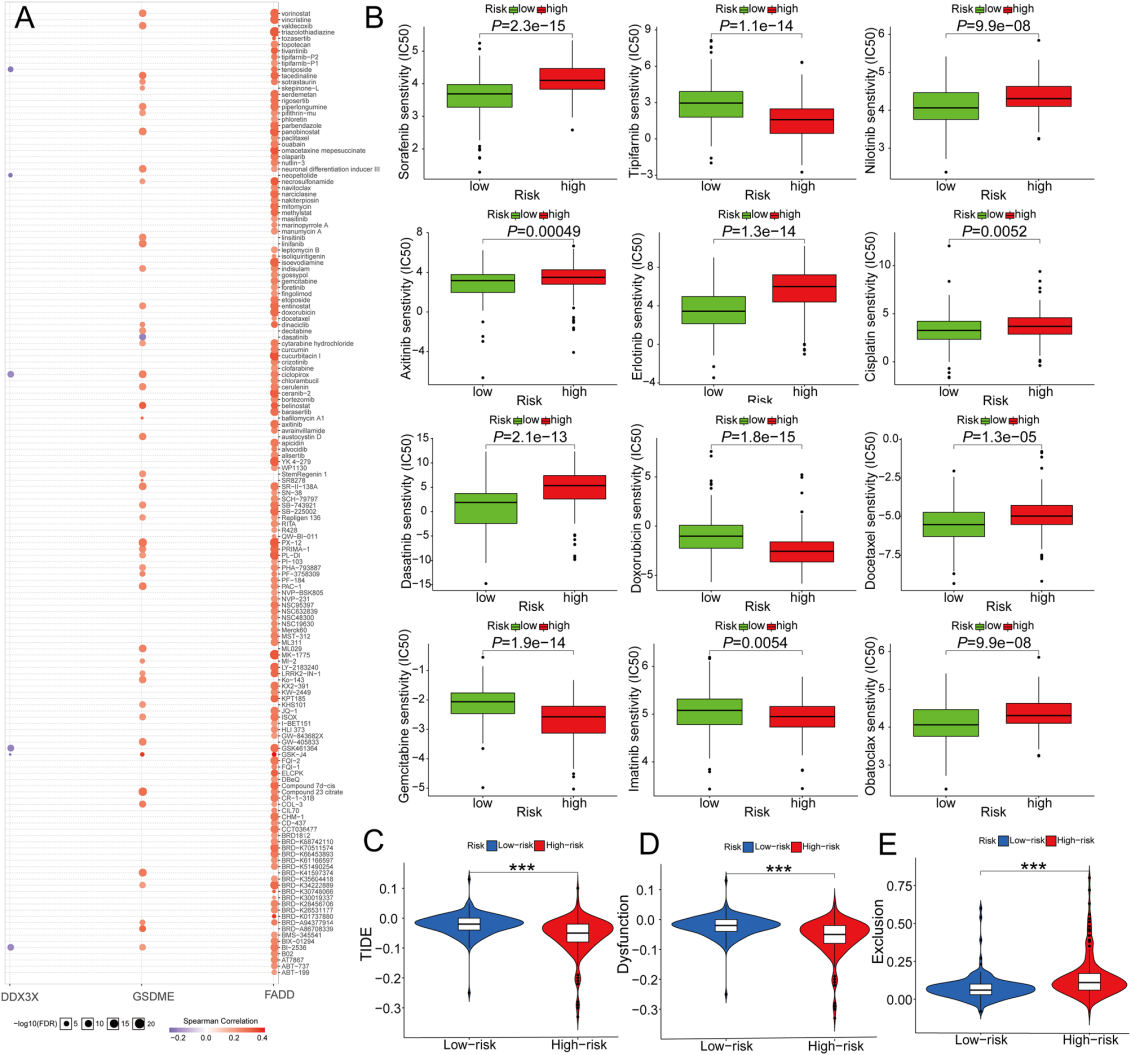
**(A**) In CTRP database, the correlations between the *FADD*, *DDX3X*, *GSDME* expression level and multiple drug sensitivity in HCC patients were discovered. Red indicates a positive correlation (the higher gene expression level carries the greater drug sensitivity), while blue indicates vice versa. The size represents the strength of drug targeting. (**B**) Drug sensitivity analysis of specific chemotherapeutics and targeted therapy agents for HCC showed that eight medications (Sorafenib, Nilotinib, Axitinib, Erlotinib, Dasatinib, Cisplatin, Docetaxel, and Obatoclax) displayed significantly higher IC50 in high-risk patients, whereas Tipifarnib, Imatinib, Doxorubicin, and Gemcitabine had significantly lower IC50, suggesting that the patients with high PANRG-score were more susceptible to Tipifarnib, Imatinib, Doxorubicin, and Gemcitabine. (**C**–**E**) The calculated scores of TIDE, immune dysfunction, and immune exclusion were compared between the high- and low-risk subgroups. The result is that the high-risk patients had markedly lower TIDE and immune dysfunction scores but higher immune exclusion scores. *** *P* < 0.001. CTRP, Cancer Therapeutics Response Portal; TCGA, the Cancer Genome Atlas database; HCC, hepatocellular carcinoma; IC50; half-maximal inhibitory concentrations; TIDE, Tumor Immune Dysfunction and Exclusion.

In single-sample Gene Set Enrichment Analysis (ssGSEA) of TCGA-HCC cohort, the high-risk subgroup exhibited much lower immune cell infiltration levels of mast cells, NK cells and pDCs, but higher levels of aDCs, iDCs and Treg than those in the low-risk subgroup. For immune-associated functions, the high-risk subgroup displayed the higher activity of MHC class I, but the lower activity of cytolytic activity, type I and II IFN Reponses (Fig. 7A,B). In the ICGC-HCC cohort, these findings were partially verified (Fig. 7C,D). In addition, the negative correlation between tumor mutation burden (TMB) and PANRG-score was revealed by spearman’s correlation analysis and the negative correlations between TMB and *DDX3X*, *CASP7*, *GSDME* expression levels were also uncovered (Fig. 7E–H, all *P* < 0.005). In Cancer Therapeutics Response Portal (CTRP) database, we noticed the *GSMDE, FADD* and *DDX3X* expression levels were positively corelated with the multiple drugs sensitivity in HCC patients, while there was no significant correlation result of *SCAF11* or *CASP7* (Fig. 8A). Using “pRRophetic” R package, the drug sensitivity analysis of specific chemotherapeutics and targeted therapy agents for HCC showed that eight medications (Sorafenib, Nilotinib, Axitinib, Erlotinib, Dasatinib, Cisplatin, Docetaxel, and Obatoclax) displayed the significantly higher half-maximal inhibitory concentrations (IC50) in the high-risk patients, whereas Tipifarnib, Imatinib, Doxorubicin, and Gemcitabine had significantly lower IC50, which suggested that the patients with high PANRG-score are more likely susceptible to Tipifarnib, Imatinib, Doxorubicin, and Gemcitabine (all *P* < 0.05, 8B). A computationally based online program called Tumor Immune Dysfunction and Exclusion (TIDE; http://tide.dfci.harvard.edu/) can stimulate body immune system and forecast an immunotherapy response. High TIDE could indicate non-responders whose suppressive cells inhibit T cell infiltration. We observed that the high-risk patients had markedly lower TIDE and immune dysfunction scores but higher immune exclusion scores than those of the low-risk patients (all *P* < 0.001, 8C-E).

### Validating the gene expression of *FADD* and exploring the correlation between PANoptosis and ferroptosis, cuproptosis

**Figure 9:**
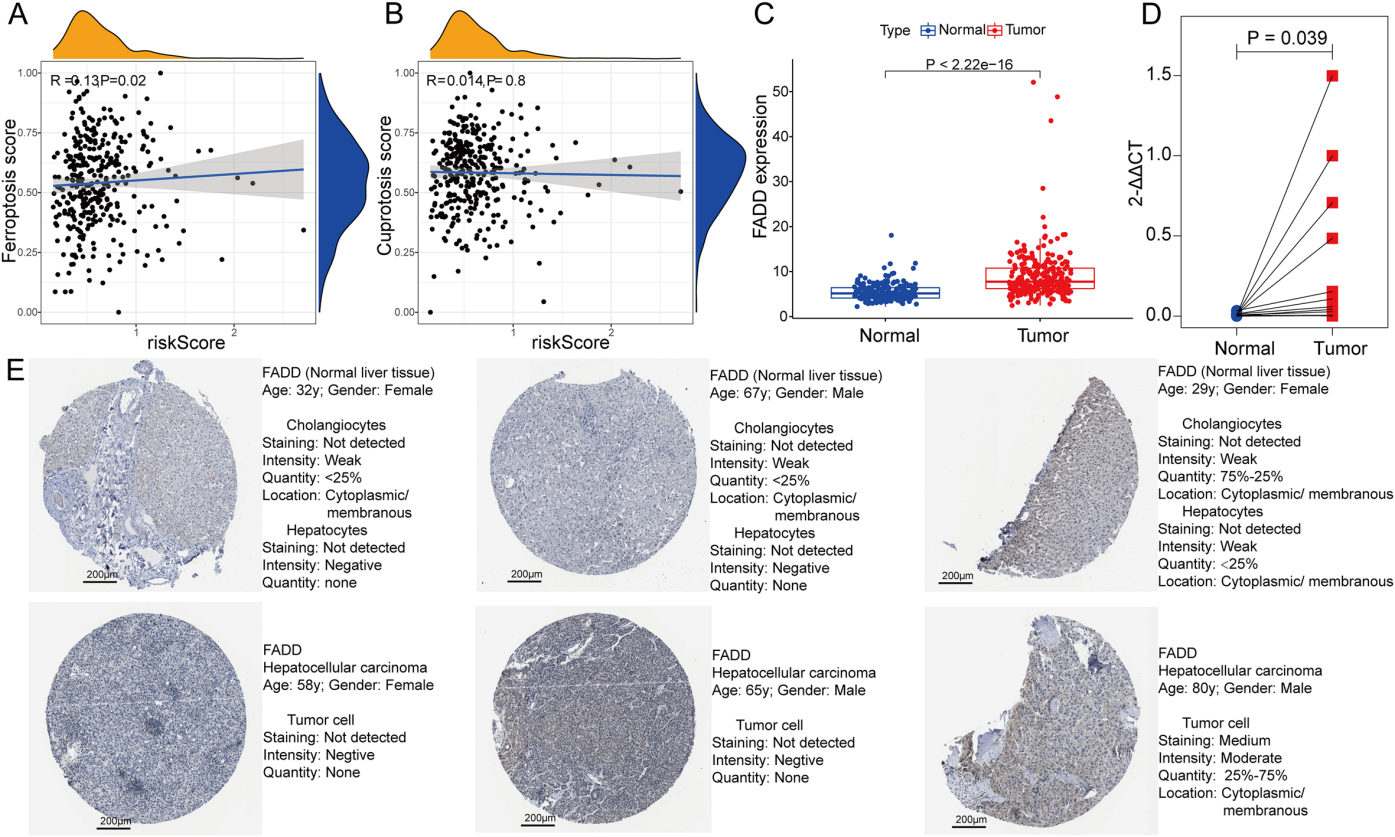
(**A**,**B**) The correlation analyses were conducted between the PANRG-score and the enrichment score of ferroptosis (**A**) and cuproptosis (**B**) respectively in the TCGA-HCC cohort. The results showed a significantly negative correlation between PANRG-score and the calculated ferroptosis ES (R = -0.13, *P* < 0.05). (**C**, **D**) The augmented expression of *FADD* level in HCC tissue was further verified in the ICGC and Guangxi cohorts. (**E**) Representative immunohistochemistry results of the normal liver and HCC tissues representing the protein expression level of *FADD* gene were explored in the HPA database. PANRG, PANoptosis-related gene; TCGA, the Cancer Genome Atlas database; HCC, hepatocellular carcinoma; ES, enrichment score; ICGC, International Cancer Genome Consortium.

Ferroptosis and cuproptosis are the novel forms of PCD and are promising as the momentous targets for HCC therapeutics^26, 27^. Therefore, we investigate the potential connection between PANoptosis and ferroptosis, cuproptosis. In Spearman analysis, the negative association between PANRG-score and the calculated ES of ferroptosis was significant (Fig. 9A; R = -0.13, *P* < 0.05). However, the cuproptosis ES and the PANRG-score do not significantly correlate (Fig. 9B). Finally, *FADD*, as an element of PANRG-score signature, one of the hub genes of PPI network on differentially expressed PANRGs, and the gene related to multiple medicines, is demonstrated to be a master regulator in HCC development^28^. In the ICGC-HCC and Guangxi cohort, the *FADD* gene expression level, compared with normal liver tissue, was also considerably augmented in HCC tissue (Fig. 9C,D). However, there is no clear difference at the protein level (Fig. 9E).

## Discussion

PCD refers to an activated death process in the innate immune system and aberrant physiological states or diseases, which sustains the balance of the internal environment^29^. The bidirectional co-ordinations between different PCDs have been revealed. For example, *FADD* and caspase-8 were discovered to be the stimulations of *NLRP3* inflammasomes, which demonstrated the connection between apoptotic and pyroptosis processes^30^. Furthermore, Taabazuing CY et al. demonstrated that the apoptotic caspase-3 and 7 pointedly block pyroptosis by cleaving *GSDMD* from the inflammatory caspases^31^. Liproxstatin-1 (LPT1), a ferroptosis inhibitor, can defend against steatosis and steatohepatitis in the mice with metabolic associated fatty liver disease and PANoptosis may be involved in regulatory process^32^. Consistently, our result also detected the potential connection between PANoptosis and ferroptosis. One of the characteristics of cancer in an immunosuppressive tumor microenvironment is blocking cell death. Tumor cells frequently have innate resistance in the apoptotic pathways, despite the fact that many therapy regimens targeting apoptotic pathways achieve good clinical effectiveness^8^. Therefore, to surmount this nature of malignant cell, the novel cancer therapeutic approaches targeting additional cell death pathways have constantly emerged. Activating “PANoptosis” could overcome resistance to apoptosis by intensely triggering inflammatory cell death^13^. Moreover, it also implies that PANoptosis holds potential to kill cancer cells directly^22^. In fact, moderate PANoptosis benefits cancer patients by inducing immune cells infiltration to inhibit viral infection or malignancy development, while inordinate PANoptosis may induce detrimental inflammation and tissue damage.

For liver cancer, cell death can cause inflammation, fibrosis, and angiogenesis, all of which are closely modulated by a range of resident and infiltrating host cells^7^. Besides, inflammasomes have emerged as pivotal innate sensors, which have a strong pathogenicity in a variety of liver illnesses. The accumulation of inflammatory substances will accelerate the progression from liver cirrhosis to primary liver cancer by promoting cellular stress, damage, and transformation. For instance, activation of the *NLRP3* inflammasome can lead to hepatocyte pyroptosis, liver inflammation, and liver fibrosis in mice^33^, in which *DDX3X*, a driving factor of *NLRP3* inflammasome, may regulate live-or-die cell-fate decisions under stress condition^34^. Toll-like receptor-mediated innate immune stimulation reduces the necessity for RIPK1 kinase activity and it can trigger the onset of the NLRP3 inflammasome and PANoptosis when TAK1 is immobilized^35^. Therefore, targeting hepatocellular cell death may be able to prevent chronic inflammatory liver disease from progressing to fibrosis and even malignancy.

In our study, multiple PANGRs had abnormal expressions in HCC. The prognostic value of these regulatory genes for HCC patients was demonstrated. They may be predominantly involved in the signal pathways associated with cell death and cancer development. Caspases are putatively considered to regulate immune reaction, homeostasis, and cell death^3, 36, 37^. Caspase-8 is essential for PANoptosis, which can promote T cell-mediated immunity and inhibit tumor growth when it comes to cancer. By activating Caspase-8, the executor caspase-3 and caspase-7 can be removed from the downstream proteins^37^. Besides, members of the Gasdermin family have a momentous impact on the pyroptosis activation and the formation of plasma membrane pore. Additionally, suppressing *SCAF11* expression level could inhibit proliferation, attenuate migration and induce apoptosis in the liver cancer cell lines^38^. The studies of these sensors and signaling molecules establish the basis for us to quest the significance of immune-mediated PANoptosis in HCC and also provide desirable targets for therapeutic intervention^5^.

In the comprehensive HCC treatment, monoclonal and bispecific antibodies targeting inhibitory pathways such as programmed cell death-1(PD-1), programmed cell death ligand 1 (PD-L1) and CTL antigen 4 (CTLA-4) have been demonstrated to activate the cytokines and cytotoxic mechanisms of adaptive immunity to override the resistance to cell death, eradicate cancer cells, and improve overall prognosis^22, 28, 39, 40^. In our study, we observed a comparatively low immune cell infiltration and immunological response function in the high PANRG-score subgroup, which may support the conclusion that the high-risk subgroup had a poor prognosis. In the drug sensitivity analysis, the high-risk subgroup exhibited the markedly lower TIDE and immunological dysfunction score, which may respond better when receiving immune checkpoint inhibitors. Carina Hage et al. demonstrated that sorafenib promoted pyroptosis in macrophages and stimulated cytotoxicity mediated by natural killer cells in HCC^41^. Besides, by increasing the cellular reactive oxygen species (ROS) levels, the synergistic effect of cysteine desulfurase deficiency and oxaliplatin can induce PANoptosis in *vitro* and *vivo* colorectal cancer model^42^. In addition, as an activator of PANoptosis, interferon regulatory factor 1 can induce inflammatory cell death during colitis-associated tumorigenesis^20^. PANoptosis could mitigate cell death-related drug resistance by enhancing local inflammation.

This research is subject to several limitations. Firstly, our PANoptosis-related risk scoring system should be further explored by biological experiments and validated in multicenter studies. Additionally, to further prove the result, the vitro experiments to verify our drug sensitivity results should be performed in our next studies. Finally, TIDE score is a computational framework for evaluating the likelihood of tumor immune escape based on gene expression profile of tumor samples, which represents tumor immune dysfunction and exclusion function. Actually, with the rise of immunotherapy in HCC, deeper researches, managing to acquire individualized TIDE score based on our indigenous and clinical HCC sample, are worth carrying out to identify HCC patients who could benefit from anti-PD-1/ PD-L1/CTLA-4 immunotherapy.

In conclusion, we set up a PANGR-score risk signature to provide a roadmap for HCC patient stratification and predict patients' prognosis. Patients with the higher PANRG-score may have a dismal survival and barren immune infiltration, but a potentially better immunotherapy response. Nevertheless, further experimentation should be conducted to establish the compliance of PANoptosis-related risk scoring system. Certainly, future therapy perspectives of HCC should emphasize the setting of PANoptosis to achieve a personalized, practicable and effective therapeutic regimen.

## Materials and methods

### Datasets and preprocessing

RNA sequencing gene expression profile (FPKM value), somatic mutation and corresponding clinical information were retrieved via the Cancer Genome Atlas (TCGA) dataset (https:// portal.gdc.cancer.gov/repository). As external validation cohorts, we collected data on microarray expression profiling and corresponding survival information from the International Cancer Genome Consortium (ICGC) and the Gene Expression Omnibus (GEO) database https://dcc.icgc.org/projects/LIRI-JP and https://www.ncbi.nlm.nih.gov/geo/, ID: GSE14520, GSE10186). Patients with follow-up periods of fewer than 30 days and incomplete clinicopathological information were removed. Furthermore, the HCC patients who had surgery at the First Affiliated Hospital of Guangxi Medical University provided sixteen pairs of tissue samples as the Guangxi cohort.

### Identification of differentially expressed PANRGs

The heatmap-visualized differentially expressed PANRGs were screened via the "limma" R package. The location of the differentially expressed PANRGs on the chromosome were visualized using the "Rcircos" R package. The PPI network were analyzed by the Search Tool for the Retrieval of Interacting Genes website (STRING v11.0, https://string-db.org/). Therein, the threshold cutoff value of interaction coefficient was 0.4^43^.

### Mutation and bioinformatics analysis of PANRGs

The landscape and oncoplot waterfall plot of gene mutation for the TCGA-HCC dataset were illustrated utilizing the “maftools” R package. Using the cBioPortal for Cancer Genomics online platform (http://www.cbioportal.org/), the genetic alterations and mutation frequencies of the differentially expressed PANRGs were also explored. To pinpoint their prospective molecular mechanisms and essential biological characteristics, the GO and KEGG analyses were carried out using the “Bioconductor” and “org.Hs.eg.db” R packages. The “GOplot” package was used to develop the visualization of significant enrichment terms with both adjusted *P* and q-value < 0.05.

### Consensus clustering analysis

Depending on consensus clustering algorithm and the similarities of PANRGs expression profiles, we applied the "ConsensusClusterPlus" R package to classify the TCGA-HCC patients (1,000 times repetitions, resample rate of 80%, and Pearson correlation). The clinicopathological characteristics of different clustering groups were compared. To determine the appropriate number of clusters to ensure the stability of the PANoptosis-related clustering pattern, the k-means approach in consensus clustering analysis was adopted^44^. Meanwhile, survival and PCA analysis between different clusters were also performed.

### Establishment, assessment, and validation of PANRGs-related prognostic risk model

Firstly, the prognosis-related genes were screened from PANRGs using univariate Cox regression analysis. Candidate genes need to meet two conditions, namely both *P* and KM value < 0.05. Furthermore, the LASSO Cox regression analysis was undertaken to prevent the hazard of overfitting using the "survival" and "glmnet" R packages. Therein, the penalty parameter(λ) was determined according to the minimum criteria. The PANRGs-score risk model was developed incorporating the genes that were still significant after screening and their accompanying regression coefficients. The calculated median PANRG-score was used to categorize the subgroups as high- and low-risk. Kaplan–Meier and ROC curves were drawn to evaluate the prognostic significance of model. Additionally, we also verified if the various clinicopathological characteristics and the PANRG-score connected. Based on the formula of our signature, the GSE14520 and GSE10186 and ICGC cohorts were stratified by the respective median of calculated PANRG-score to further validate the prognostic value of PANoptosis-related risk scoring system. Then, to evaluate the clustering capability, PCA and t-SNE analyses was applied.

### Construction and validation of a predictive nomogram

To find the independent prognostic predictor, univariate and multivariate Cox regression analyses of PANRG-score and clinical info were implemented. Using the "rms" R package, a nomogram with the PANRG-score and easily accessible and widely accepted clinicopathological parameters (gender, age, histologic grade, and pathological stage) was established to forecast the OS for patients with HCC. The bootstrap method was applied to calculate concordance index (C-index) with 1000 resamples. The discrimination capability was examined based on the fitting degree of calibration curves. The "DynNom" and "shiny" R packages were utilized to generate the online version of nomogram and uploaded on the "shinyapps" website.

### GSEA and immune infiltration analysis

In the TCGA and ICGC datasets, the immune-associated cell infiltration levels and function pathways between high- and low-risk subgroups were analyzed and compared by ssGSEA algorithm of the "gsva" and "limma" R package. In the GSEA software program (v4.0.1; 1,000 permutations), the significant enrichment levels of biological GO and KEGG function pathway between different subgroups was analyzed based on the TCGA-HCC transcriptome data and the gene sets "c5.all.v7.1.symbols.gmt" and "c2.cp.kegg.v7.0.symbols.gmt" for reference. The term with both the false discovery rate (FDR) and nominal *P* < 0.05 was believed to be a statistically significant pathway. As is known, the distribution density of non-synonymous mutations in the protein coding region were quantified as TMB. The correlation between the TMB of TCGA-HCC sample and corresponding PANRG-score was investigated by spearman’s correlation analysis.

### Drug sensitivity prediction analysis

To figure out the IC50 of particular chemotherapeutics and targeted therapy agents for HCC, we utilized the “pRRophetic” R package which is based on ridge regression method and the comprehensive data from Genomics of Drug Sensitivity in Cancer (GDSC; www.cancerrxgene.org/) and TCGA database^45^. The online Gene Set Cancer Analysis (GSCALite; http://bioinfo.life.hust.edu.cn/web/GSCALite/) platform provides the comprehensive analysis of genomic cancer, in which the drug IC50 data and cancer cell line expression spectrum based on CTRP database were integrated^46^. Based on the TCGA-HCC cohort, the calculated TIDE, immune dysfunction, and immune exclusion scores can be acquired from the TIDE website and were further compared between the different PANRG-score subgroups.

### Correlation between ferroptosis, cuproptosis and PANoptosis

The relationship between PANRG-score and the enrichment score (ES) of ferroptosis and cuproptosis, which were figured out by the gene-set-based Gene Set Variation Analysis (GSVA) approach, were analyzed using Spearman method. Therein, the genes for ferroptosis pathway were extracted from the Molecular Signatures Database (https://www.gsea-msigdb.org/gsea/msigdb) and the cuproptosis-related genes were collected from the prior literature^47, 48^.

### Polymerase chain reaction (PCR) and HPA database

In Guangxi cohort, the differences of *FADD* gene expression level between HCC and neighboring non-cancerous tissues were compared using paired t-tests. Our prior study has expounded how to preserve specimens and the concrete steps for RNA extraction and reverse transcription-quantitative PCR^49^. The primer sequences for PCR of reference and *FADD* were as follows: GAPDH, forward: GTCAGCCGCATCTTCTTT, reverse: CGCCCAATACGACCAAAT. *FADD*, forward: GAATCGGAGCGAAGCAGAGA, reverse: ACCCTAGTGTCCAGGTCTGT. The mRNA expression levels of *FADD* were measured by 2^−ΔΔCT^ method. Furthermore, the *FADD* protein expression level in the normal liver and HCC tissue was explored using Human Protein Atlas database (HPA; https://www.proteinatlas.org).

### Statistical analysis

R (v4.1.1) was employed to perform data analysis and result visualization. The Wilcoxon rank-sum test was performed between the groups of continuous data, while the chi-square test was used for categorical data. The Kaplan–Meier method with log-rank test was utilized to conduct survival analysis. The hazard ratios of inclusion factors and their corresponding 95% confidence intervals in the Cox regression analysis were calculated. Unless otherwise indicated, *P* < 0.05 was provided as the threshold cutoff value.

### Ethical approval

TCGA, GEO and ICGC datasets used human genomic data deposited in public repositories, so ethics approval is not applicable to these datasets. For the Guangxi cohort, the experimental protocol was established, according to the ethical guidelines of the Helsinki Declaration and this study was approved by the Ethics Committee of the First Affiliated Hospital of Guangxi Medical University. All patients provided written informed consent prior to being operation on.

### Supplementary Information

Supplementary Figures.

Supplementary Tables.

## Data Availability

TCGA gene expression profile (FPKM value), somatic mutation and corresponding clinical information during the current study are publicly available in the TCGA-LIHC repository (https:// portal.gdc.cancer.gov/repository). The data on microarray expression profiling and corresponding survival information from the ICGC and the GEO database are publicly available in ICGC data portal, and GSE14520 and GSE10186 repository (https://dcc.icgc.org/projects/LIRI-JP and https://www.ncbi.nlm.nih.gov/geo/). The original contributions presented in the study are included in the article. Further inquiries can be directed to the corresponding author.

## Abbreviations

AUC: Area under curve
C-index: Concordance index
CNV: Copy number variations
CTLA-4: CTL antigen 4
CTRP: Cancer therapeutics response portal
ES: Enrichment score
FDR: False discovery rate
GDSC: Genomics of drug sensitivity in cancer
GEO: Gene expression omnibus
GO: Gene oncology
GSCA: Gene set cancer analysis
GSEA: Gene set enrichment analysis
GSVA: Gene set variation analysis
HCC: Hepatocellular carcinoma
HPA: Human protein atlas database
IC50: Half-maximal inhibitory concentrations
ICGC: International Cancer Genome Consortium
KEGG: Kyoto encyclopedia of genes and genomes
LASSO: Least absolute shrinkage and selection operator
LPT1: Liproxstatin-1
OS: Overall survival
PANRGs: PANoptosis-related genes
PCA: Principal component analysis
PCD: Programmed cell death
PCR: Polymerase chain reaction
PD-1: Programmed cell death-1
PD-L1: Programmed cell death ligand 1
PPI: Protein–protein interactions
ROC: Receiver operating characteristic
ROS: Reactive oxygen species
SNP: Single nucleotide polymorphisms
ssGSEA: Single-sample gene set enrichment analysis
STRING: Search tool for the retrieval of interacting genes
TCGA: The cancer genome atlas
TIDE: Tumor immune dysfunction and exclusion
TMB: Tumor mutation burden
t-SNE: T-distributed stochastic neighbor embedding
