# Supplementary material for: Robust analysis of a novel PANoptosis-related prognostic gene signature model for hepatocellular carcinoma immune infiltration and therapeutic response
Source: Sci Rep. 2023 Sep 4;13:14519. doi: 10.1038/s41598-023-41670-9 (PMC10477271; doi:10.1038/s41598-023-41670-9)
Supplement: Supplementary file 1 — Supplementary Figures. [file 41598_2023_41670_MOESM1_ESM.docx]

**Supplementary Files**

**Figures and Figure Legends**


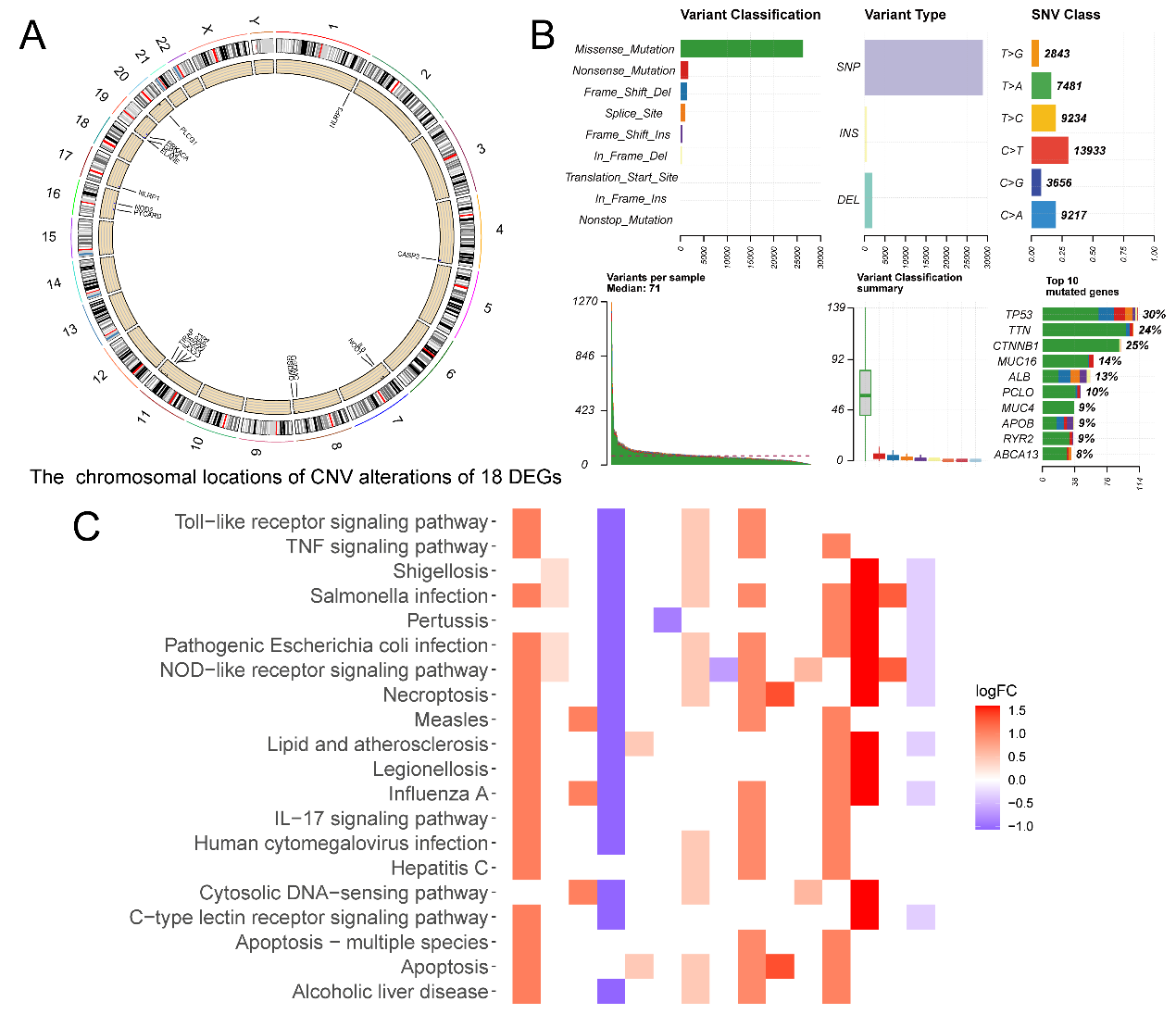
 Supplementary Figure S1. (A) Circus plots depicts the chromosome distributions of differentially expressed PANRGs. (B) The landscape of mutation profiles of the TCGA-HCC patients, in which TP53 mutation was the most prevalent mutation in HCC. (C) The enriched item in KEGG analysis shows that the differentially expressed PANRGs were significantly correlated with the pathways related to HCC carcinogenesis, cell death and liver diseases. Red indicates positive correlation, while blue indicates negative correlation. The intensity of the colors represents the strength of relevance. PANRGs, PANoptosis-related genes; TCGA, the Cancer Genome Atlas database; HCC, hepatocellular carcinoma; TP53, Tumor protein p53; KEGG, Kyoto Encyclopedia of Genes and Genomes.


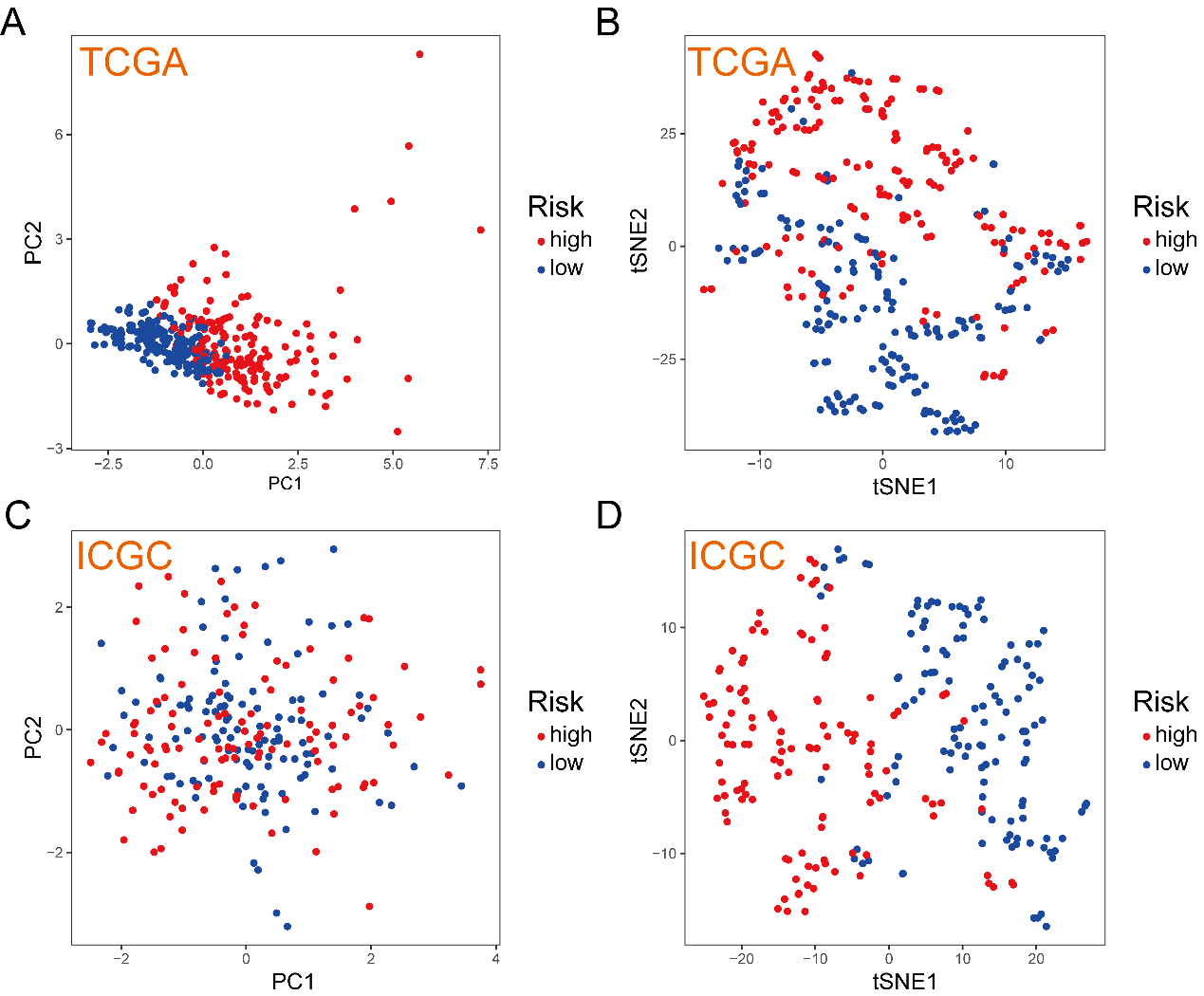


Supplementary Figure S2. PCA and tSNE analyses revealed an effective clustering ability of PANRG-score risk signature in the TCGA (A, B) and ICGC (C, D) cohorts. PCA, Principal component analysis; tSNE, t-distributed stochastic neighbor embedding; TCGA, the Cancer Genome Atlas database; ICGC, International Cancer Genome Consortium database.


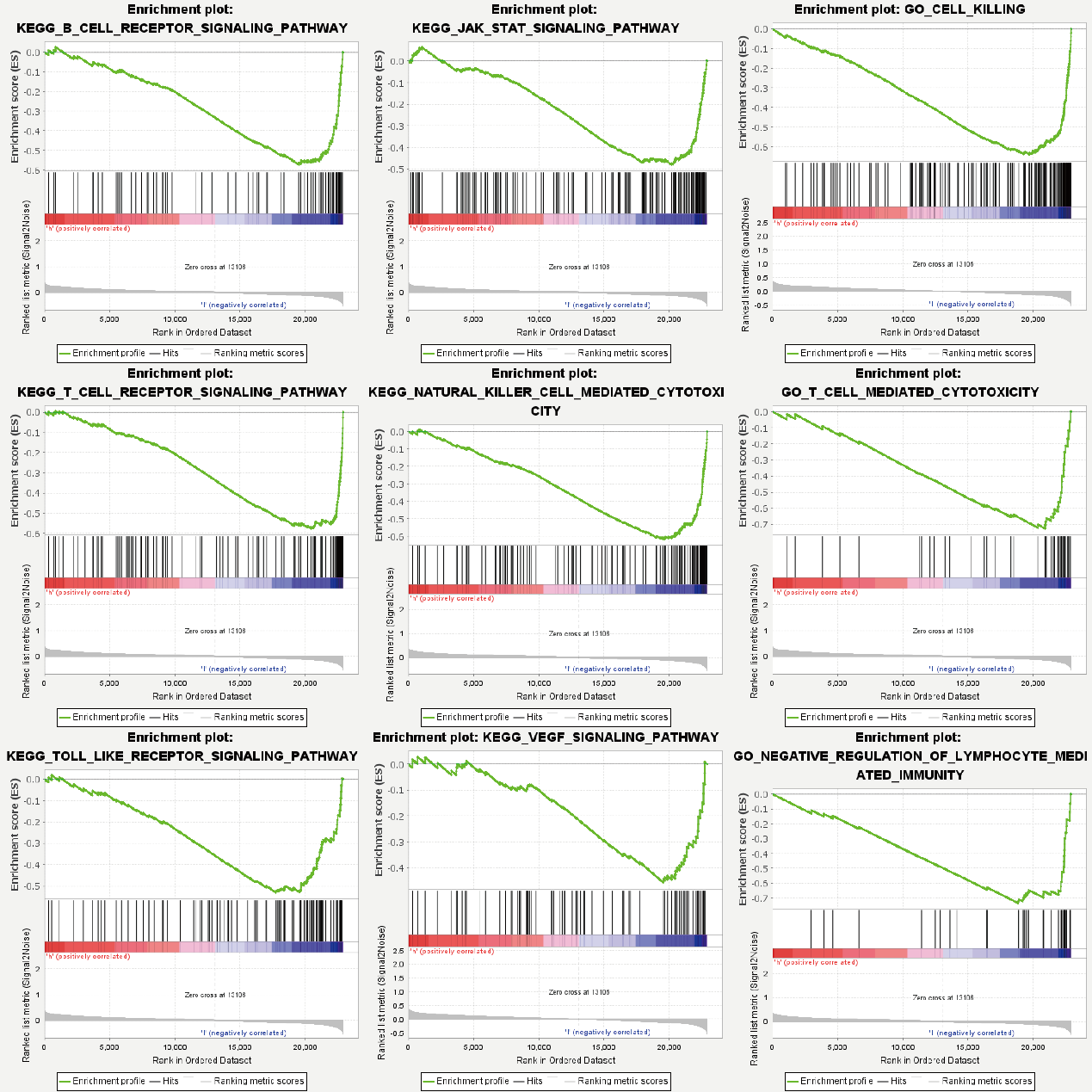


Supplementary Figure S3. The results of function enrichment analysis using the GSEA software program show that multiple cell death- and cancer-related pathways were considerably abundant in the low-risk subgroup.


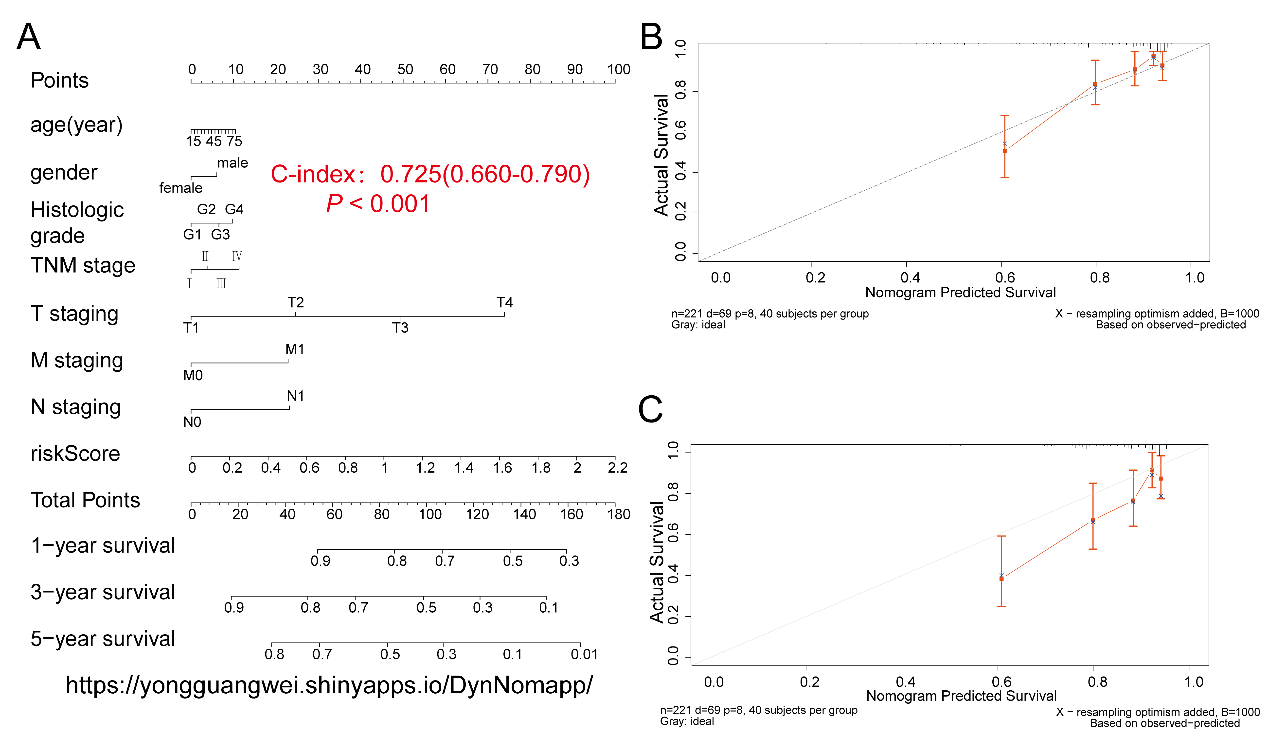


Supplementary Figure S4. (A) A prognostic nomogram combining the PANRG-score and clinicopathological parameters was built to determine the likelihood of the 1-, 3-, and 5-year OS in TCGA-HCC patients. (B, C) Calibration plot was developed to prove the predicting probability of our nomogram for at 1- and 2- year OS of TCGA-HCC patients.


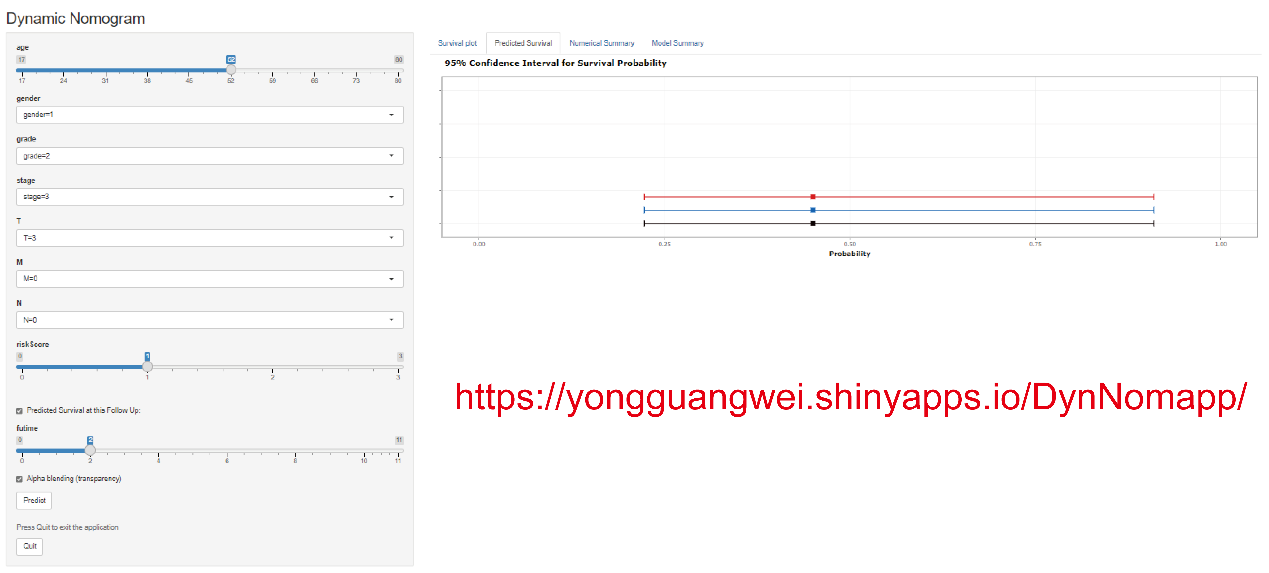


Supplementary Figure S5. The web-based calculator of established nomogram featuring PANRG-score and clinicopathological parameters was used to predict the OS of specific HCC patients expediently. Gender: 0 (female) and 1 (male); Grade, histologic grade (1-4: Grade 1-4); Stage, TNM staging (1-4: Stage Ⅰ-Ⅳ); T, T staging (1-4: T1-T4 staging); N, N staging (0-1: N0-N1 staging); M, M staging (0-1: M0-M1 staging).
