# Supplementary material for: Robust analysis of a novel PANoptosis-related prognostic gene signature model for hepatocellular carcinoma immune infiltration and therapeutic response
Source: Sci Rep. 2023 Sep 4;13:14519. doi: 10.1038/s41598-023-41670-9 (PMC10477271; doi:10.1038/s41598-023-41670-9)
Supplement: Supplementary file 2 — Supplementary Tables. [file 41598_2023_41670_MOESM2_ESM.docx]

**Supplementary Tables**

Supplementary Table S1. The important PANoptosis-related genes (PANRGs) extracted from previous articles.

| Gene | Description | Chromosome Location |
| --- | --- | --- |
| *AIM2* | absent in melanoma 2 | Chromosome 1, NC_000001.11 (159059226..159132351, complement) |
| *PYCARD* | *PYD* and *CARD* domain containing | Chromosome 16, NC_000016.10 (31201486..31202760, complement) |
| *CASP3* | caspase 3 | Chromosome 4, NC_000004.12 (184627696..184649447, complement) |
| *CASP8* | caspase 8 | Chromosome 2, NC_000002.12 (201233443..201287711) |
| *RIPK1* | receptor interacting serine/threonine kinase 1 | Chromosome 6, NC_000006.12 (3063967..3115187) |
| *FADD* | Fas associated via death domain | Chromosome 11, NC_000011.10 (70203296..70207390) |
| *GSDME* | gasdermin E | Chromosome 7, NC_000007.14 (24698355..24762235, complement) |
| *CASP7* | caspase 7 | Chromosome 10, NC_000010.11 (113679194..113730909) |
| *CASP1* | caspase 1 | Chromosome 11, NC_000011.10 (105025443..105035591, complement) |
| *CASP11* | caspase 11 | Chromosome 12, NC_000012.12 (45919131..45992040, complement) |
| *TAK1* | *TGF*-beta activated kinase 1 | Chromosome 3, NC_000003.12 (14947583..15049273) |
| *MLKL* | mixed lineage kinase domain like pseudokinase | Chromosome 16, NC_000016.10 (74671855..74700889, complement) |
| *GSDMD* | gasdermin D | Chromosome 8, NC_000008.11 (143553387..143563062) |
| *NLRP3* | *NLR* family pyrin domain containing 3 | Chromosome 1, NC_000001.11 (247416163..247448823) |
| *ZBP1* | Z-DNA binding protein 1 | Chromosome 20, NC_000020.11 (57603852..57620480, complement) |
| *TNF* | tumor necrosis factor | Chromosome 6, NC_000006.12 (31575565..31578336) |
| *IFNG* | interferon gamma | Chromosome 12, NC_000012.12 (68154768..68159740, complement) |
| *ADAR* | adenosine deaminase RNA specific | Chromosome 1, NC_000001.11 (154582057..154627997, complement) |
| Gene | Description | Chromosome Location |
| *IL1B* | interleukin 1 beta | Chromosome 2, NC_000002.12 (112829751..112836843, complement) |
| *NLRC4* | *NLR* family *CARD* domain containing 4 | Chromosome 2, NC_000002.12 (32224449..32265743, complement) |
| *IRF8* | interferon regulatory factor 8 | Chromosome 16, NC_000016.10 (85899162..85922609) |
| *NAIP* | *NLR* family apoptosis inhibitory protein | Chromosome 5, NC_000005.10 (70968166..71025339, complement) |
| *CASP4* | caspase 4 | Chromosome 11, NC_000011.10 (104942866..104968596, complement) |
| *CASP5* | caspase 5 | Chromosome 11, NC_000011.10 (104994243..105023168, complement) |
| *IL18* | interleukin 18 | Chromosome 11, NC_000011.10 (112143251..112164104, complement) |
| *NLRP12* | *NLR* family pyrin domain containing 12 | Chromosome 19, NC_000019.10 (53793584..53824403, complement) |
| *DDX3X* | *DEAD*-box helicase 3 X-linked | Chromosome X, NC_000023.11 (41333308..41364472) |
| *CASP6* | caspase 6 | Chromosome 4, NC_000004.12 (109688628..109703445, complement) |
| *PARP1* | Poly (ADP-ribose) polymerase 1 | Chromosome 1, NC_000001.11 (226360691..226408093, complement) |

Supplementary Table S2. The differentially expressed PANoptosis-related genes (PANRGs) in HCC based on TCGA database.

| Gene* | conMean | treatMean | logFC | *P* |
| --- | --- | --- | --- | --- |
| *AIM2* | 0.250 | 0.378 | 0.598 | 0.01 |
| *PYCARD* | 3.242 | 9.797 | 1.596 | <0.001 |
| *CASP3* | 4.186 | 8.441 | 1.012 | <0.001 |
| *CASP8* | 1.377 | 2.831 | 1.040 | <0.001 |
| *RIPK1* | 6.726 | 9.203 | 0.452 | <0.001 |
| *FADD* | 2.299 | 4.460 | 0.956 | <0.001 |
| *GSDME* | 0.422 | 1.127 | 1.419 | <0.001 |
| *SCAF11* | 3.500 | 4.376 | 0.322 | 0.007 |
| *NR2C2* | 0.656 | 1.528 | 1.220 | <0.001 |
| *GSDMD* | 12.953 | 31.075 | 1.262 | <0.001 |
| *NLRP3* | 0.352 | 0.279 | -0.334 | 0.002 |
| *ADAR* | 16.433 | 33.481 | 1.027 | <0.001 |
| *IL1B* | 0.874 | 0.418 | -1.064 | <0.001 |
| *IRF8* | 5.601 | 3.002 | -0.900 | <0.001 |
| *CASP4* | 4.201 | 5.107 | 0.282 | 0.033 |
| *NLRP12* | 0.040 | 0.025 | -0.682 | <0.001 |
| *CASP6* | 5.030 | 6.864 | 0.448 | <0.001 |
| *PARP1* | 7.888 | 20.100 | 1.349 | <0.001 |

* The corresponding description of genes can be found in the Supplementary Table S1.
